# US trade policy and public health: heterogeneous effects from the North American Free Trade Agreement

**DOI:** 10.1017/S1368980024001472

**Published:** 2024-09-18

**Authors:** Derick T. Adu, Wendiam P.M. Sawadgo, Wenying Li

**Affiliations:** 1 Department of Agriculture, Alcorn State University, Lorman, MS, USA; 2 Department of Agricultural Economics and Rural Sociology, Comer Hall, Auburn University, Auburn, AL, USA

**Keywords:** North American Free Trade Agreement, Sugar trade agreement, Diabetes, Difference-in-differences, Event study

## Abstract

**Objective:**

To investigate the causal link between the North American Free Trade Agreement (NAFTA) unrestricted sugar trade agreement signed in 2008 between the USA and Mexico and the diabetes prevalence across all fifty US states.

**Design:**

A quasi-experimental research design to investigate the causal effect of the NAFTA unrestricted sugar trade agreement on diabetes prevalence. Our study utilises a comprehensive panel dataset spanning from 2000 to 2016, comprising 1054 observations. To conduct our analysis, we applied both the difference-in-differences and event-study methodologies.

**Setting:**

All the states in the USA.

**Participants:**

The fifty states in the USA.

**Results:**

After the enactment of the NAFTA sugar trade agreement between the USA and Mexico in 2008, most states witnessed an increase in diabetes prevalence. The annual impacts displayed significant variation among states, with percentage increases spanning from 0·50 to 2·28 %.

**Conclusions:**

States with a higher percentage of their population living below the poverty line, a larger Black resident population and a lower proportion of high school graduates had more significant increases in diabetes prevalence attributed to the NAFTA sugar trade agreement.

A bilateral sugar trade agreement was established between Mexico and the USA in 2008 under the North American Free Trade Agreement (NAFTA). This agreement removed restrictions on the flow of sugar between these countries^(1)^. By 2013, Mexico became responsible for 66 % (equivalent to two million short tonnes in raw value) (see Fig. 1) of the total sugar imports to the USA, which led to a sharp decline in domestic sugar prices and an increase in the average annual consumer surplus to nearly $1·67 billion^(2)^. Diabetes prevalence in the USA went up instantaneously after 2008^(3)^. Between 2008 and 2012, crude diabetes prevalence increased from 7·3 to 10·1 % (see the Health Care Cost Institute, 2013). In this paper, we study the impact of the NAFTA sugar provision on diabetes prevalence and evaluate which states in the USA were most affected by the policy.

**Fig. 1:**
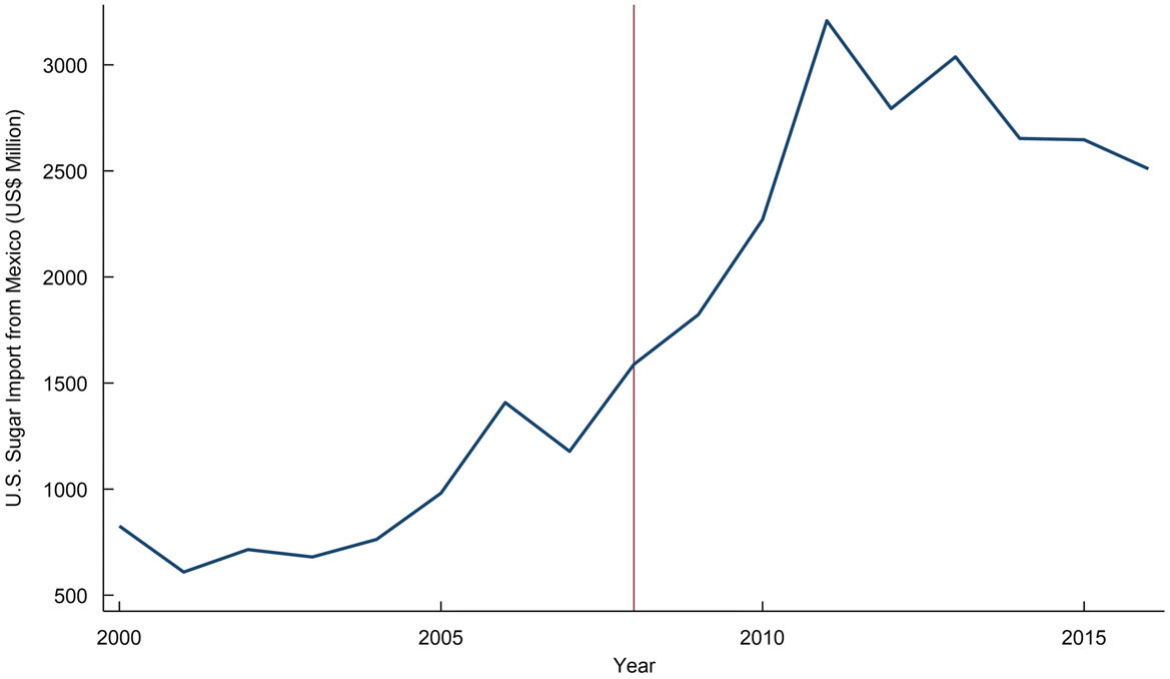
Trend of US sugar import from Mexico

Governments around the world have had increased interest in global public health in recent years. Many health experts and economists have turned their attention to the link between sugar consumption and diseases like obesity and type 2 diabetes. For instance, type 2 diabetes, dental caries and obesity are all associated with the intake of sugar-sweetened beverages (SSB)^(4)^. Furthermore, the increase in the consumption of foods with high sugar content has been shown to increase age-standardised BMI by 0·033 kg/m^2(5)^. As a result, the WHO^(6)^ put out an initiative to reduce added-sugar intake with the goal of improving global public health. Governments throughout the world have responded by establishing programmes like sugar taxes to lower the amount of sugar in food and drinks^(7)^. Policymakers in the UK, for example, introduced a two-tier soft drink industry levy to reduce sugar intake, successfully reducing childhood obesity^(8,9)^. Studies have found SSB taxes to reduce purchases of taxed sugar beverages in Mexico^(10)^, Berkeley, California^(11)^ and the Netherlands^(12)^, a finding that has been supported by several meta-analyses^(13)^. Sugar taxes have also been shown to reduce diseases associated with SSB intake, such as obesity and type 2 diabetes^(14–16)^.

Despite the widely recognised advantages of free trade agreements, which include economic development, lower government spending, technology transfer, enforcing competition among firms, enhancing productivity and reducing prices and markups^(17,18)^, their possible unintended effects on public health are often ignored. The removal or reduction of sugar trade barriers between partner countries increases imports while decreasing commodity prices, essentially having the opposite effect of a sugar tax. Four pathways were identified by^(19)^ through which trade openness affects health: higher imports and immigration, agricultural commodities trade, labour markets and structural adjustment measures. Prior work has shown that the unhindered trade in commodities like cigarettes, alcohol and ultra-processed foods poses risks to public health^(20–22)^. For example, a 10 % rise in trade openness generates a 0·8 % increase in obesity rates^(20)^. Since sugar trade liberalisation behaves as a subsidy, making more sugar available for consumption in the recipient country, this poses a risk to public health^(21,22)^. As a result, considerable evaluation and steps are required to offset any adverse health effects from trade agreements.

Our primary objective in this study is to analyse the potential causal effects of NAFTA’s unrestricted sugar trade agreement on diabetes prevalence across the fifty US states. While free trade agreements provide enormous benefits, it is critical to recognise their unintended implications on public health. To our knowledge, only two studies^(22,23)^ have examined the direct health implications associated with trade agreements. In^(23)^ ‘selected countries’, free trade agreements with the USA and their impact on obesity in the USA are studied. Whereas^(23)^ investigates free trade agreements more broadly, our study is distinct by focusing specifically on the potential causal impact of NAFTA’s sugar agreement. Our study is similar to^(22)^ which investigates the causal impact of the NAFTA sugar trade agreement on diabetes prevalence using the synthetic control method. However, whereas^(22)^ focused on the USA as a whole, we employ the difference-in-difference (DD) and event-study approaches to examine the causal impact of the NAFTA sugar trade agreement on diabetes prevalence in each of the fifty US states. In doing so, we explore which populations were the most adversely affected by the policy since sugar consumption patterns and economic and sociodemographic characteristics vary from state to state.

To achieve our main objective, we use a DD research design – a quasi-experimental method commonly employed to evaluate the effects of policies or programmes following^(24,25)^ and others. The DD estimate allows us to compare the differences in outcomes before and after the treatment (difference one) between a group exposed to the treatment and a control group (difference two). Additionally, we conducted a panel event study, where the ‘event’ was the date of implementation of the NAFTA sugar agreement following^(25)^. Our findings provide compelling evidence supporting concerns that the unrestricted sugar trade agreement under NAFTA poses a risk to public health. We observed an increase in diabetes prevalence ranging from 0·54 to 2·3 % across various states within the USA following the implementation of the trade agreement. We find that states with a higher percentage of their population below the poverty level, a greater percentage of the Black population and a lower percentage of high school graduates were associated with greater increases in diabetes prevalence as a result of the NAFTA sugar trade agreement.

## Pathways of trade liberalisation and public health

Trade policies have a substantial impact on power dynamics, wealth distribution and resource allocation, which influence working conditions, health choices and overall well-being^(26)^. When trade liberalisation is well-executed, it can boost economic growth by expanding export and investment options. In theory, this can help alleviate poverty and promote human health by improving the economic stability, labour standards, access to affordable healthcare and nutrition^(27)^. Poorly implemented trade policies and agreements, on the other hand, have been proven to heighten power, money and resource distribution inequality between and within nations, having a negative impact on health and health equality^(28)^.

Increased trade and investment in health-harming goods like tobacco, alcohol, sugar, SSB and highly processed foods have occurred concurrently with the rise of free trade agreements, which demand changes in domestic policies and regulatory frameworks^(29)^. This has resulted in the spread of unhealthy lifestyles throughout the world. The prevalence of diabetes, obesity and diet-related noncommunicable diseases has significantly increased over the past couple of decades, particularly in low- and middle-income countries. Rates of obesity and noncommunicable diseases in low- and middle-income countries are now equal to or higher than those in high-income countries^(30)^.

The transformation of global food systems can be linked to the opening of domestic markets for international food trade, the increased involvement of transnational food corporations, the rise in foreign direct investment in the food industry and the extensive global marketing and promotion of food products^(31)^. Food trade patterns have shifted, increasing trade volumes for hazardous foods while lowering trade volumes for conventional cereals and starchy root crops^(32)^. Following NAFTA, countries such as Mexico experienced significant US agribusiness investment, reshaping domestic agriculture into export-oriented cash crop production, and that affected the availability of food, quality of nutrition, price and desirability^(32)^. Similarly, in Central America and Asia, decreased investment barriers contributed to the rise of highly processed food markets and lower regulatory standards in the food business^(33)^. Furthermore, attempts to create a health-based labelling system for snack products in Thailand faced criticisms from the USA and other countries, impacting the final decision on policy^(34)^. Transnational corporations owned by Americans brought the majority of these food products to Thailand^(35)^.

## Methods

To identify the causal effect of the NAFTA sugar agreement on diabetes prevalence, we employ a DD model with fixed effects and an event study. The primary limitation to using DD analysis and randomised control trials in macroeconomic research is the challenge of meeting key assumptions, such as the ‘parallel trend assumption’. This assumption, which requires that the trends in the treatment and control groups would have been similar in the absence of the treatment, is difficult to establish at the macroeconomic level. To address these concerns, we rigorously tested the parallel trend assumption to ensure the validity of our estimates following^(36)^. The panel event-study method establishes causal relationships by comparing outcomes before and after an event. It effectively controls time-invariant heterogeneity and time trends, allowing for a flexible time specification. However, it relies on the assumption of common trends and can be sensitive to the choice of time periods. Additionally, its findings may have limited generalisability and require substantial data. Despite these limitations, it provides valuable insights into the dynamic effects of events on outcomes^(37)^. In our research design, a treated state adopted the NAFTA sugar agreement in the USA, while a control region (i.e. the control country includes six Organisation for Economic Co-operation and Development countries, including Australia, China, Norway, Japan, Switzerland and the UK) adopted no such trade policy. Intuitively, the idea is to compare the difference in average diabetes prevalence between treated and control regions before and after the treatment. The data analysis was performed using Stata 18 software and packages such as ‘did’, ‘hdid’ and ‘eventdd’, as well as Stata’s built-in regression functionality for linear regression.

### The difference-in-differences analysis

We estimate the following equation:
(1)

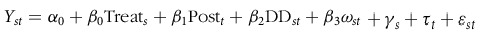

in which 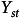 is a continuous variable indicating crude diabetes prevalence in state 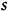 at year 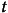 , 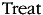 is a binary variable indicating whether the state (or country) had adopted NAFTA, 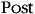 is a binary variable taking a value of 1 in the post-NAFTA period (and 0 otherwise) and *DD* is the interaction between 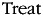 and 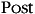 . Our parameter of interest is 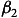 . Finally, 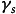 and 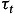 are, respectively, state and year fixed effects. Including year and country fixed effects is crucial for controlling time-specific and country-specific factors affecting diabetes prevalence. Year fixed effects account for time trends, while country fixed effects control for country-specific characteristics. This reduces omitted variable bias, improves comparability across countries and over time and provides more accurate estimates of variable relationships. However, our analysis captures unaccounted time-varying factors by using fixed effects at the country and time level. For example, differences in diabetes screening across countries or over time are captured through the fixed effects. However, if unobserved factors vary significantly across different states and these variations have different temporal trends, our model may not fully account for their impacts. This limitation suggests that the fixed effects model could underestimate the influence of these unobserved variables, which may vary not only by state but also over time. Future research might use models such as mixed-effects models or the inclusion of additional observable covariates to better control these factors. The variable 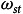 captures variables such as the population aged above 65 years, the log of raw sugar import (metric tonnes) and the percentage of the female population, which have been found to affect diabetes prevalence in prior studies^(22)^. Studies such as those by^(22,38,39)^ have shown that an increase in sugar imports results in higher consumption of sugar and SSB, leading to a rise in diabetes and other diseases. Research also indicates that the prevalence of diabetes varies by age group, with older adults having the highest prevalence among all age groups^(40–42)^. Additionally, studies have shown that diabetes prevalence varies by gender, with a higher prevalence among females than males^(43)^. To overcome possible serial correlation problems, we used a robust se following^(44)^. The key identifying assumption of DD analyses is that of common trends between treated states and the control countries in the absence of the treatment. We tested the parallel trend assumption following^(25)^ and removed states that did not meet the assumption from the analysis. We employed a Wald test, comparing against a null hypothesis of zero, to evaluate whether the linear trends were parallel before the treatment^(25)^.

#### Event-study analysis

In addition to the DD analysis described above, we conducted a panel event study, with the ‘event’ being the date of implementation of the NAFTA sugar trade agreement in a particular state. We estimated the following equation:
(2)

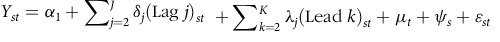

where 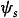 and 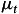 are binary variables for state and year and 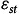 is the unobserved error term. Further, 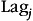 and 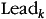 are two binary variables indicating the number of years until the implementation of the NAFTA sugar agreement in state 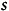 . Formally, we defined 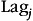 and 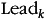 according to equations (3)–(6):
(3)

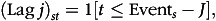



(4)

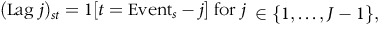



(5)

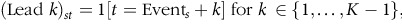



(6)

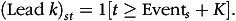

where 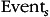 is a variable indicating the year 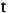 in which the NAFTA sugar agreement was implemented in state 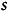 . The first 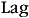 was omitted to capture the baseline difference between the treated and control state.

#### Effect heterogeneity over time

To assess how the effects of the NAFTA sugar agreement vary with time, we follow^(45)^ to estimate a dynamic model where 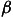 can vary across years:
(7)

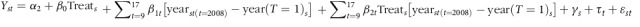




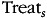
 is a binary variable equal to 1 if State 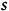 is ever treated (i.e. part of the NAFTA sugar agreement). Then, 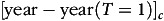 is the difference between the observation year and the first year of implementation of the NAFTA sugar trade agreement in State 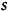 . The parameters of interest are the 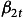 , which represents the mean difference in the crude diabetes prevalence in a specific year 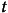 . We also control for State 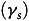 and year 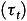 fixed effects.

## Data

**Fig. 2:**
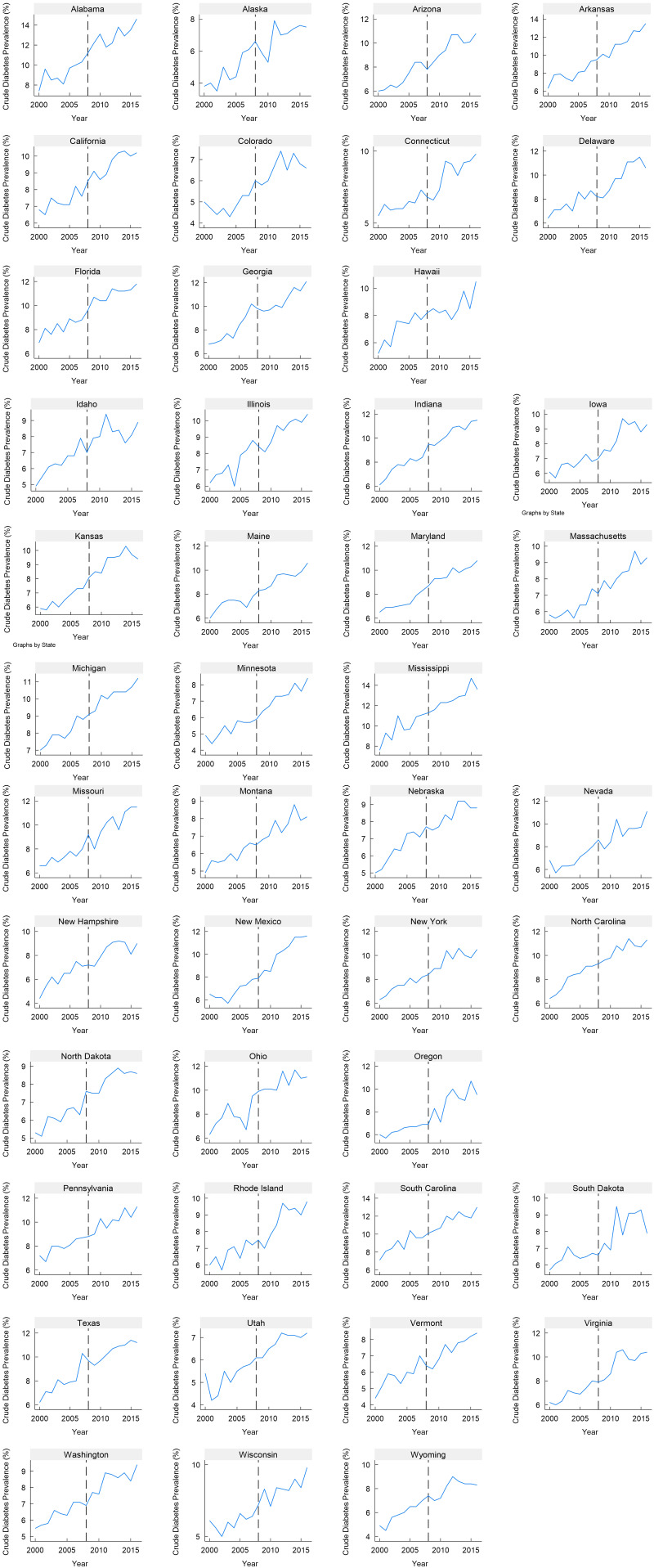
Trends of diabetes prevalence by states

**Fig. 3:**
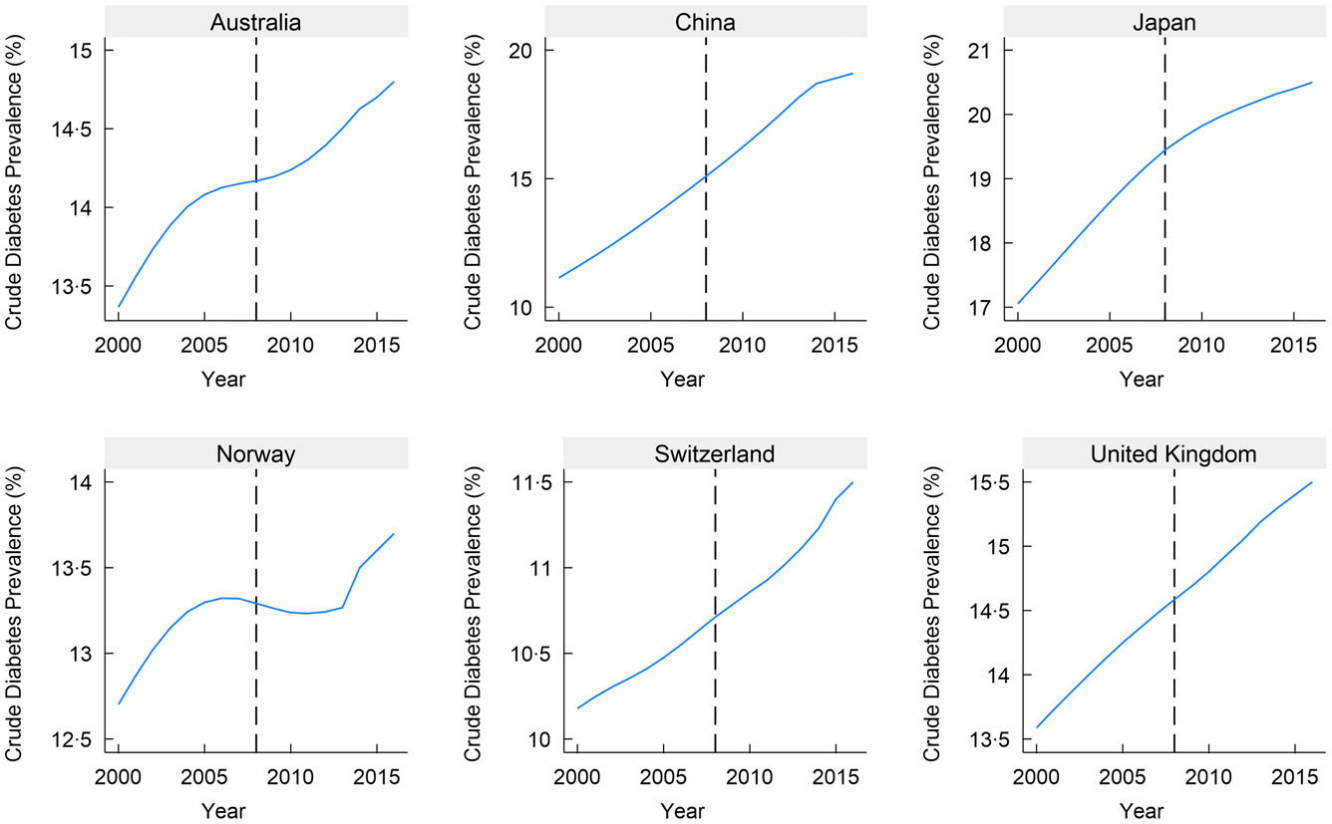
Trends of diabetes prevalence by control countries

Our analysis is conducted using a panel dataset that includes data at both the state and country levels from 2000 to 2016. The dataset contains 1054 observations, covering the fifty US states and six countries from the Organisation for Economic Co-operation and Development each year between 2000 and 2016. Data on crude diabetes prevalence that comprises type 1 and type 2 diabetes both at the state and country levels were obtained from the US Diabetes Surveillance website maintained by the Centers for Disease Control and Prevention and the NCD Risk Factor Collaboration (NCD-RisC). Data on sugar imports were sourced from the Food and Agriculture Organization of the United Nations Statistics Office (FAOSTAT). We obtained data on the percentage of poverty (all ages) from the 2009 US Census Bureau, Small Area Estimates Branch. The percentage of the Black population was obtained from the 2009 Census Bureau. Gender-related data were acquired from Kaiser Family Foundation (KFF) estimates, which relied on the 2008–2021 American Community Survey’s 1-Year Estimates. Additionally, data on educational attainment (high school diploma or more) were sourced from the US Census Bureau’s 2009 American Community Survey. Although a stronger strategy would concentrate solely on type 2 diabetes, which is affected by dietary modifications, we faced a lack of precise data regarding type 2 diabetes, particularly for the control countries. Considering the worldwide escalation in diabetes occurrence primarily linked to type 2 diabetes^(46)^, we hold the viewpoint that incorporating the combined prevalence of type 1 and type 2 diabetes will not impact the fundamental argument of this research. Figures 2 and 3 illustrate the diabetes trend for the states and countries used in our analysis.

## Results

**Fig. 4:**
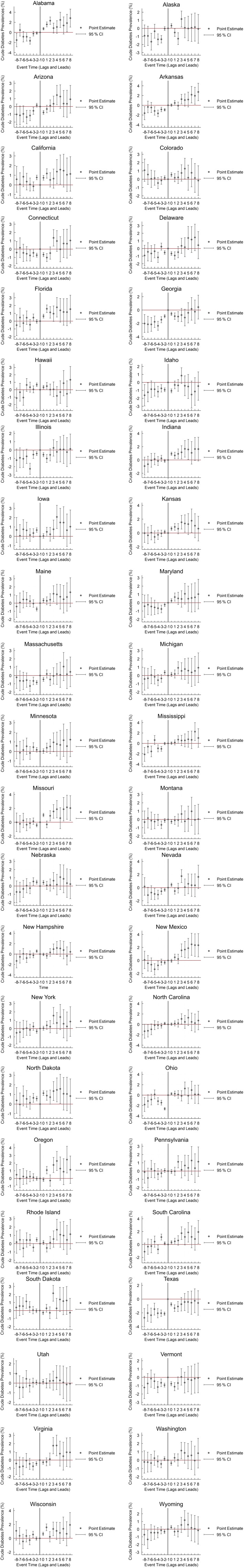
Event-study results

In our study, we investigate the causal impact of NAFTA’s sugar trade agreement on diabetes prevalence in the fifty US states. Overall, we find that the NAFTA sugar trade agreement has significant positive impacts on diabetes prevalence in most of the fifty states (see Fig. 4 and Table 1). Figure 4 displays the results of the event-study analysis, while Table 1 shows the estimated average treatment effects from the DD analysis. Across most states, both the DD and event analysis methods revealed significant effects of the NAFTA sugar trade agreement on diabetes prevalence. Because the NAFTA sugar trade agreement was a national policy, one would expect the states to experience similar effects. However, the impact on the fifty states varies. The estimated effect varies in statistical significance and magnitude from 2·28 % (*P* < 0·001) (95 % CI from 1·54 to 3·02) in Alabama to 0·50 % (*P* < 0·1) (95 % CI from −0·07 to 1·07) in Iowa (Table 1).

**Table 1:** Difference-in-differences (DD) estimated results by state

State	DD estimates	95 % CI		Parallel trend (*P* value)
Alabama	2·280**	1·542	3·019	0·35
Alaska	1·18**	0·25	2·111	0·909
Arizona	1·175***	0·512	1·838	0·606
Arkansas	1·074*	–0·007	2·154	0·92
California	0·768***	0·28	1·256	0·236
Colorado	0·261	–0·272	0·795	0·141
Connecticut	0·68**	0·028	1·333	0·792
Delaware	0·799	0·099	1·5	0·262
Florida	1·246***	0·757	1·735	0·723
Georgia	1·113***	0·424	1·803	0·124
Hawaii	0·258	–0·437	0·954	0·812
Idaho	0·372	–0·242	0·986	0·1
Illinois	0·669**	0·062	1·277	0·132
Indiana	1·446*	0·952	1·94	0·183
Iowa	0·501*	–0·068	1·071	0·606
Kansas	1·217***	0·748	1·685	0·69
Maine	0·736***	0·237	1·235	0·68
Maryland	1·049	0·622	1·475	0·769
Massachusetts	0·725***	0·211	1·239	0·982
Michigan	0·733***	0·289	1·178	0·473
Minnesota	0·503**	0·036	0·969	0·724
Mississippi	1·471***	0·703	2·238	0·941
Missouri	0·963**	0·002	1·924	0·486
Montana	0·289	–0·177	0·755	0·934
Nebraska	0·572**	0·055	1·089	0·11
Nevada	1·089***	0·439	1·738	0·626
New Hampshire	0·709**	0·001	1·416	0·169
New Mexico	1·297**	0·229	2·364	0·234
New York	0·808***	0·304	1·313	0·473
North Carolina	1·012***	0·439	1·585	0·1
North Dakota	0·739**	–0·027	1·504	0·607
Ohio	1·437***	0·706	2·169	0·746
Oregon	1·008***	0·33	1·686	0·596
Pennsylvania	0·708***	0·189	1·228	0·606
Rhode Island	0·500*	–0·096	1·096	0·346
South Carolina	1·224***	0·548	1·899	0·12
South Dakota	0·249	–0·433	0·931	0·262
Texas	1·175***	0·513	1·837	0·826
Utah	0·097	–0·391	0·585	0·596
Vermont	0·232	–0·279	0·742	0·431
Virginia	1·165***	0·557	1·773	0·5
Washington	0·550**	0·059	1·041	0·667
Wisconsin	0·869***	0·296	1·442	0·303
Wyoming	0·712***	0·185	1·24	0·135

Note: ***P < 0.01, **P < 0.05, *P < 0.1.

We classified the fifty states by the magnitude and significance of the estimated impact of the NAFTA sugar agreement on diabetes prevalence. We assessed the parallel trend assumption as per Shahid *et al.* (2022) and excluded states that did not fulfil this assumption from the analysis. Our estimates suggest that two states (Alabama and Arkansas) saw crude diabetes prevalence increase by greater than two percentage points as a result of the NAFTA sugar agreement. Fifteen states had an impact greater than 1 % but less than 2 % (Alaska, Arizona, Florida, Georgia, Indiana, Kansas, Mississippi, Nevada, New Mexico, North Carolina, Ohio, Oregon, South Carolina, Texas and Virginia). Eighteen states saw less than a 1 % impact (California, Connecticut, Illinois, Iowa, Maine, Massachusetts, Michigan, Minnesota, Missouri, Nebraska, New Hampshire, New York, North Dakota, Pennsylvania, Rhode Island, Washington, Wisconsin and Wyoming). Nine states (Colorado, Delaware, Hawaii, Idaho, Maryland, Montana, South Dakota, Utah and Vermont) had no effect on diabetes prevalence from the NAFTA sugar trade agreement. This illustrates that, while most states saw a rise in diabetes prevalence because of the NAFTA sugar trade agreement, some did not. Lastly, six states (Kentucky, Louisiana, New Jersey, Oklahoma, Tennessee and West Virginia) did not meet the parallel trend assumption.

To better understand the rationale behind the disparities in trade policy impact across states, we explore the relationship between selected covariates that have been shown in the health literature to be major factors influencing diabetes prevalence. We use 2008 values of the covariates for the analyses, as this represents the midpoint of our dataset. We concentrated on poverty, educational attainment, percentage of the population that is Black and percentage of the population that is female. All these variables have a statistically significant association (*P* value < 0·001) with the estimated diabetes prevalence. Higher poverty level, for example, is associated with higher diabetes prevalence, having a greater Black population is associated with diabetes prevalence, having a lower percentage of the population with a high school degree is associated with higher diabetes prevalence and having a higher percent female population is associated with higher diabetes prevalence.

These findings are consistent with previous health research. For example, research has demonstrated that individuals with greater levels of education are more likely to practise preventative healthcare behaviours, such as eating healthier meals, exercising more and preventing type 2 diabetes and obesity^(47,48)^. Also, studies have investigated the effect of race on type 2 diabetes prevalence. The difference in type 2 diabetes prevalence across race and ethnic groups includes the prevalence of certain risk factors such as obesity and limited access to healthy foods. For example^(49)^, found that type 2 diabetes prevalence is higher in the Black population (5·97 %) compared with whites (0·77 %) in the USA from 2014 to 2015. It has also been shown that diabetes incidence has a greater link with poverty level^(50)^. Several studies have found that diabetes prevalence varies between males and females^(50)^. These findings point to the fact that the state-level differences in crude diabetes prevalence as shown in the current study can be attributed to sociodemographic characteristics such as poverty, race, gender and educational attainment.

**Fig. 5:**
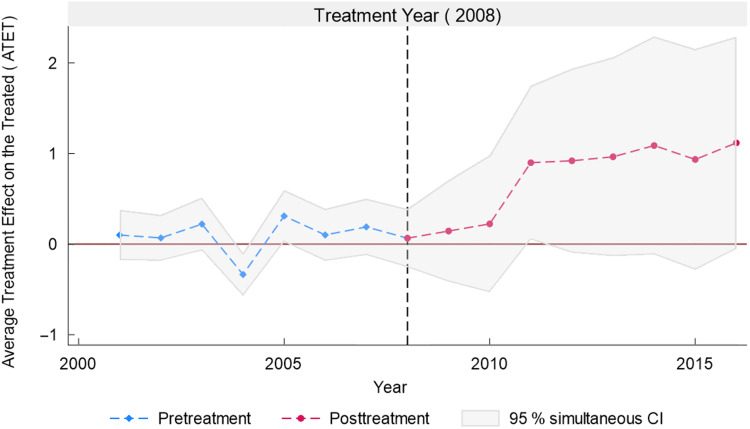
Average treatment effect on the treated in pretreatment and posttreatment years

**Fig. 6:**
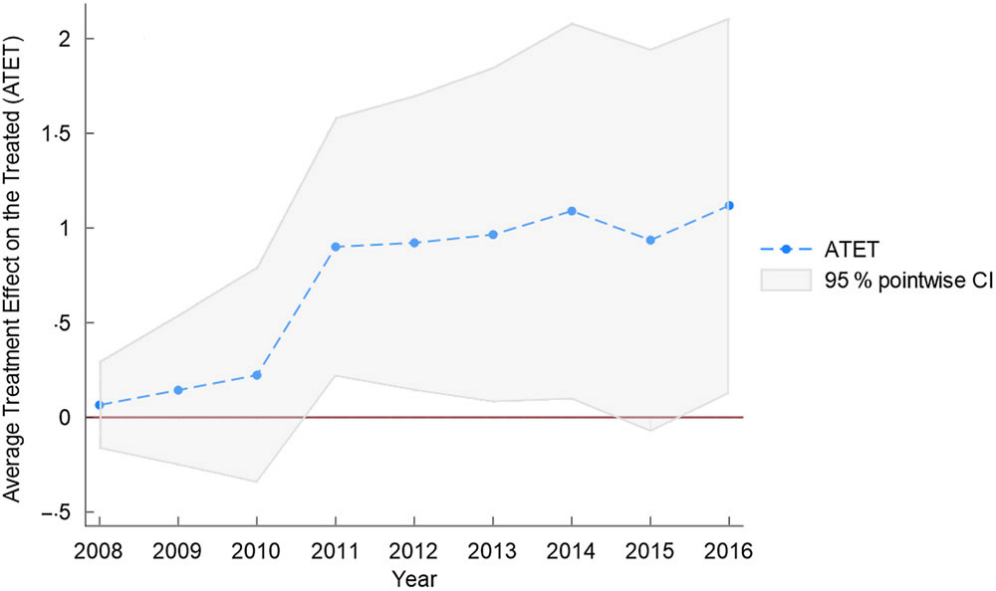
Average treatment effect on the treated in posttreatment years

**Fig. 7:**
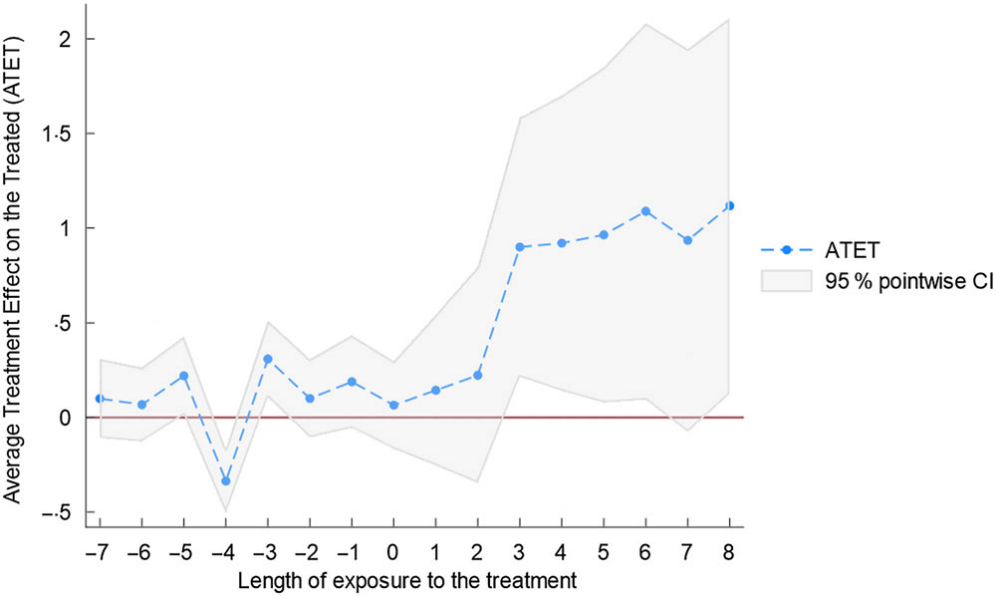
Average treatment effects on the treated over different lengths of exposure to treatment

Figures 5–7 as well as Table 2 present the results of equation (7). Table 2 shows the effect of the NAFTA sugar agreement for the fifty states across the posttreatment years. We observe that although the average treatment effect on the treated (ATET) was positive in 2008 (the treatment year) with its 95 % CI ranging from –0·17 to 0·30, it lacks statistical significance. This is not unexpected because just like any trade policy, the year of the agreement does not present a substantial impact. This is also reflected in Figs. 5–7, showing a marginal effect for 2008. We obtained statistically significant ATET (0·22 %) in 2009 with a 95 % CI ranging from 0·01 to 0·43. The ATET for 2010 is 0·31 % with 95 % CI ranging from 0·11 to 0·51. The ATET then increased from 0·92 % in 2012 (95 % CI from 0·14 to 1·70) to 1·12 % in 2016 (95 % CI from 0·13 to 2·11).

**Table 2:** Posttreatment – average treatment effect on the treated (ATET)

Year	ATET	Robust sd	*P* value	(95 % CI)
2001	0·100	0·107	0·346	(–0·108, 0·309)
2002	0·069	0·100	0·491	(–0·127, 0·264)
2003	0·145	0·203	0·477	(–0·254, 0·543)
2004	–0·336	0·087	0	(–0·506, –0·165)
2005	0·224	0·291	0·442	(–0·346, 0·794)
2006	0·101	0·106	0·338	(–0·106, 0·308)
2007	0·190	0·125	0·129	(–0·055, 0·435)
2008	0·066	0·118	0·577	(–0·166, 0·297)
2009	0·221	0·106	0·037	(0·014, 0·428)
2010	0·310	0·104	0·003	(0·107, 0·513)
2011	0·900	0·349	0·01	(0·216, 1·584)
2012	0·920	0·398	0·021	(0·141, 1·700)
2013	0·964	0·452	0·033	(0·079, 1·849)
2014	1·089	0·508	0·032	(0·094, 2·084)
2015	0·935	0·516	0·07	(–0·076, 1·946)
2016	1·118	0·507	0·027	(0·125, 2·111)

Number of observations: 952.

As a robustness check, we used equation (1) to apply the difference-in-difference approach at the aggregate level (see Table 3). Our regression model specifications revealed a statistically significant impact of the NAFTA sugar trade agreement on diabetes prevalence. This result is consistent with all our other findings.

**Table 3:** Results of difference-in-difference (DD) regression

Variables	(1)	(2)	(3)	(4)	(5)	(6)	(7)	(8)
Post	3·364***	3·474***	3·554***	2·843***	3·806***	2·948***	2·805***	3·031***
	(0·259)	(0·252)	(0·408)	(0·237)	(0·406)	(0·234)	(0·330)	(0·333)
Treat	–4·062***	–4·464***	–4·204***	–4·430***	–4·732***	–4·748***	–4·404***	–4·810***
	(0·277)	(0·288)	(0·318)	(0·264)	(0·344)	(0·273)	(0·290)	(0·309)
Post 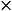 Treat	0·980***	0·882***	0·979***	0·628***	0·874***	0·558***	0·627***	0·558***
	(0·185)	(0·179)	(0·187)	(0·209)	(0·182)	(0·207)	(0·209)	(0·208)
Female population (%)		0·491***			0·524***	0·402***		0·411***
		(0·103)			(0·108)	(0·093)		(0·096)
Population aged above 65 years			–0·048		–0·082		0·009	–0·020
			(0·059)		(0·059)		(0·048)	(0·047)
Log (raw sugar import)				0·961***		0·932***	0·963***	0·926***
				(0·169)		(0·171)	(0·169)	(0·170)
Constant	12·88***	–12·20**	13·61***	6·166***	–12·63**	–14·18***	6·011***	–14·27***
	(0·194)	(5·247)	(0·877)	(1·221)	(5·321)	(4·842)	(1·413)	(4·872)
Observations	952	952	952	952	952	952	952	952
Year Fixed Effects	Yes	Yes	Yes	Yes	Yes	Yes	Yes	Yes
State Fixed Effects	Yes	Yes	Yes	Yes	Yes	Yes	Yes	Yes
R-squared	0·971	0·972	0·971	0·975	0·972	0·975	0·975	0·975

Robust se in parentheses: ***P < 0·01, **P < 0·05, *P < 0·1.

## Discussion

Our study investigated the causal impact of NAFTA’s sugar trade agreement on diabetes prevalence in the US fifty states. The findings reveal significant positive impacts of the NAFTA sugar trade agreement on diabetes prevalence in most states, as evidenced by both the DD and event analysis methods. This aligns with our expectations, considering the widespread nature of the policy. However, the impact varied across states, indicating that factors beyond the trade agreement itself may influence diabetes prevalence. The disparities in the impact of the NAFTA sugar agreement on diabetes prevalence across states highlight the importance of considering individual state characteristics. States with higher poverty levels, greater Black populations, lower educational attainment and higher proportions of females tended to experience greater impacts.

These findings are consistent with existing literature on the sociodemographic determinants of diabetes prevalence. The lack of statistical significance in the ATET for the treatment year (2008) is not surprising, as the immediate effects of trade agreements on health outcomes may take time to manifest. The significant ATET observed in subsequent years suggest a gradual increase in the impact of the NAFTA sugar agreement on diabetes prevalence, peaking in 2016. Our study has several implications for policy and future research. First, it underscores the need for tailored public health interventions at the state level to address the disparate impacts of trade agreements on health outcomes. Second, it highlights the importance of considering sociodemographic factors in health policy formulation and implementation. Lastly, our findings contribute to the growing body of literature on the health impacts of trade agreements, providing valuable insights for policymakers and researchers alike. In conclusion, our study provides robust evidence of the impact of the NAFTA sugar trade agreement on diabetes prevalence in the US fifty states. The findings underscore the complex interplay between trade policies and health outcomes, emphasising the need for nuanced policy responses to address the health implications of trade agreements.

### Conclusion

The surge in sugar consumption has been linked to numerous long-term illnesses that comprise of obesity and diabetes. In response to this worrisome trend, institutions like the WHO advocate for sugar taxes as a means to combat the issue. They urge politicians and governments to implement pricing structures aimed at discouraging the excessive consumption of sugary beverages. Hungary has embraced this approach and has already started taxing SSBs. On the other hand, the NAFTA sugar trade agreement had the opposite effect, resulting in reduced sugar prices in the USA. To better understand its effects, we use the difference-in-differences and event-study approaches to estimate the causal impact of the NAFTA sugar trade agreement on diabetes prevalence in the individual fifty states. The results revealed that the NAFTA sugar trade agreement has led to an increase in diabetes prevalence in most states, with rates ranging from 0·50 % in Iowa to 2·28 % in Alabama. Additionally, we examine the impact of the NAFTA sugar trade agreement across all fifty states over time. Notably, the policy’s significant impact began in 2009. With a 95 % CI, the ATET ranged from 0·22 % in 2009 to 1·12 % in 2016. These findings underscore the potential consequences of trade agreements on public health outcomes and emphasise the need for careful consideration of such policies in the future.

## Data Availability

Data will be made available on request.
